# Supplementary material for: Skin Bacteria Mediate Glycerol Fermentation to Produce Electricity and Resist UV-B
Source: Microorganisms. 2020 Jul 21;8(7):1092. doi: 10.3390/microorganisms8071092 (PMC7409288; doi:10.3390/microorganisms8071092)
Supplement: Supplementary file 1 [file microorganisms-08-01092-s001.pdf]

microorganisms

Supplementary Materials

Skin Bacteria Mediate Glycerol Fermentation to Produce Electricity and Resist to UV-B

Arun Balasubramaniam[1], Prakoso Adi[1], Do Thi Tra My[1], Jen-Ho Yang[1], Asy Syifa Labibah[1], Chun-Ming Huang[1,*]

1. Department of Biomedical Sciences and Engineering, National Central University, Taoyuan 32001, Taiwan.

* Correspondence: chunming@ncu.edu.tw (C.-M.H); Tel.: +886-3-422-7151 x 36104; Fax: +886-3-425-3427

Supplementary Table S1. 16S rRNA gene sequences of bacteria in fecal samples

Colony Name	Nucleotide Sequence	Species	% Identity
**F1**	GGGCGTGGCGCGTGCTATACTGCAGTCGAACGCTTCTTTCCTCCCGAGTGC<br>TTGCACTCAATTGAAAAGAGGAGTGGCGGACGGGTGAGTAACACGTGGG<br>TAACCTACCCATCAGAGGGGGATAACACTTGGAAACAGGTGCTAATACC<br>GCATAACAGTTTATGCCGCATGGCATAAGAGTGAAAGGCGCTTTCGGGTG<br>TCGCTGATGGATGGACCCGCGGTGCATTAGCTAGTTGGTGAGGTAACGGC<br>TCACCAAGGCCACAATGCATAACCGACCTGAGAGGGTGATCGGCCACAC<br>TGGGACTGAGACACGGCCCAGACTCCTACGGGAGGCAGCAGTAGGGAAT<br>CTTCGGCAATGGACGAAAGTCTGACCGAGCAACGCCGCGTGAGTGAAGA<br>AGGTTTTCGGATCGTAAAACTCTGTTGTTAGAGAAGAACAAGGACGTTAG<br>TAACTGAACGTCCCCTGACGGTATCTAACCAGAAAGCCACGGCTAAACTA<br>CGTGCCAGCACCCGCGGGGTAATAA	*E. faecalis*	**98.82**
**F2**	GTGCATGCGGGTGCTATACATGCAGTCGAACGCTTCTTTCCTCCCGAGTGC<br>TTGCACTCAATTGGAAAGAGGAGTGGCGGACGGGTGAGTAACACGTGGG<br>TAACCTACCCATCAGAGGGGGATAACACTTGGAAACAGGTGCTAATACC<br>GCATAACAGTTTATGCCGCATGGCATAAGAGTGAAAGGCGCTTTCGGGTG<br>TCGCTGATGGATGGACCCGCGGTGCATTAGCTAGTTGGTGAGGTAACGGC<br>TCACCAAGGCCACGATGCATAGCCGACCTGAGAGGGTGATCGGCCACAC<br>TGGGACTGAGACACGGCCCAGACTCCTACGGGAGGCAGCAGTAGGGAAT<br>CTTCGGCAATGGACGAAAGTCTGACCGAGCAACGCCGCGTGAGTGAAGA<br>AGGTTTTCGGATCGTAAAACTCTGTTGTTAGAGAAGAACAAGGACGTTAG<br>TAACTGAACGTCCCCTGACGGTATCTAACCAGAAAGCCACGGCTAACTAC<br>GTGCCAGCAGCGGGCGGGTTATTA	*E. faecalis*	**100.00**
**F3**	GTGCAGTGCGCGGCTATACTGCAGTCGAACGCTTCTTTCCTCCCGAGTGCT<br>TGCTCTCTATTGAAAAGAGGAGTGGTGGATGGGTGAGTAACACGTGGGTA<br>ACCTACCCATCAGAGGGGGATAACACTTGGAAACAGGTGCTAATACCGC<br>ATAACAGTTTATGCCGCATGGCATAAAAGTGAAAGGCGCTTTCGGGTGTC<br>GCTGATGGATGGACCCGCGGTGCATTAGCTATTTGGTGAGGTAACGGCTC<br>ACCAAGGCCACAATGCATAACCGACCTGATAGGGTGATCGGCCACGCTG<br>GGACTGAAACACGGCCCAGACTCCTACAGGAGGCAGCAGTAGGGAATCT<br>TCGGATGTGGACGAAAGTCTGACCGAGCAACGCCGCGTGAGTGAAGAAG<br>GTTTTCGGATCGTAAAACTCTGTTGTTAGAGAAGAACAATGACGTTTGTAA<br>CTGAACGTCCCCTGACGGTATCTAACCTCAAAGCCACGGCCAACTACGTG<br>CCAGCAGCCCCGGTAATAAC	*E. faecalis*	**95.34**

**F4**	GTGGCGTGCGGCTGCTATACATGCAAGTCGAACGCTTTTTCTTTCACCGGA<br>GCTTGCTCCACCGAAAGAAAAGGAGTGGCGAACGGGTGAGTAACACGTG<br>GGTAACCTGCCCATCAGAAGGGGATAACACTTGGAAACAGGTGCTAATA<br>CCGTATAACAATCGAAACCGCATGGTTTCGGTTTGAAAGGCGCTTTTGCGT<br>CACTGATGGATGGACCCGCGGTGCATTAGCTAGTTGGTGAGGTAACGGCT<br>CACCAAGGCAACGATGCATAGCCGACCTGAGAGGGTGATCGGCCACATT<br>GGGACTGAGACACGGCCCAAACTCCTACGGGAGGCAGCAGTAGGGAATC<br>TTCGGCAATGGACGCAAGTCTGACCGAGCAACGCCGCGTGAGTGAAGAA<br>GGTTTTCGGATCGTAAAACTCTGTTGTTAGAGAAGAACAAGGATGAGAGT<br>AAAATGTTCATCCCTTGACGGTATCTAACCAGAAAGCCACGGCTAACTAC<br>GTGCCAGCAGGGGCGGGGTAATAAA	***E. favium***	**99.41**

Supplementary Table S2. 16S rRNA gene sequences of bacteria in skin swabs

Colony Name	Nucleotide Sequence	Species	% Identity
**S1**	GGGGAATGGCGGGTGCTATACATGCAGTCGAGCGAACAGACG<br>AGGAGCTTGCTCCTCTGACGTTAGCGGCGGACGGGTGAGTAAC<br>ACGTGGATAACCTACCTATAAGACTGGGATAACTTCGGGAAAC<br>CGGAGCTAATACCGGATAATATATTGAACCGCATGGTTCAATA<br>GTGAAAGACGGTTTTGCTGTCACTTATAGATGGATCCGCGCCG<br>CATTAGCTAGTTGGTAAGGTAACGGCTTACCAAGGCAACGATG<br>CGTAGCCGACCTGAGAGGGTGATCGGCCACACTGGAACTGAG<br>ACACGGTCCAGACTCCTACGGGAGGCAGCAGTAGGGAATCTTC<br>CGCAATGGGCGAAAGCCTGACGGAGCAACGCCGCGTGAGTGA<br>TGAAGGTCTTCGGATCGTAAAACTCTGTTATTAGGGAAGAACA<br>AATGTGTAAGTAACTATGCACGTCTTGACGGTACCTAATCAGA<br>AAGCCACGGCTAACTACGTGCCAGCAGCCGCGGTA	***S. epidermidis***	**100.00**
**S2**	CGGCCTGCGCTGCTATACATGCAAGTCGAGCGAACAGACGAG<br>GAGCTTGCTCCTCTGACGTTAGCGGCGGACGGGTGAGTAACAC<br>GTGGATAACCTACCTATAAGACTGGGATAACTTCGGGAAACCG<br>GAGCTAATACCGGATAATATATTGAACCGCATGGTTCAATAGT<br>GAAAGACGGTTTTGCTGTCACTTATAGATGGATCCGCGCCGCA<br>TTAGCTAGTTGGTAAGGTAACGGCTTACCAAGGCAACGATGCG<br>TAGCCGACCTGAGAGGGTGATCGGCCACACTGGAACTGAGAC<br>ACGGTCCAGACTCCTACGGGAGGCAGCAGTAGGGAATCTTCCG<br>CAATGGGCGAAAGCCTGACGGAGCAACGCCGCGTGAGTGATG<br>AAGGTCTTCGGATCGTAAAACTCTGTTATTAGGGAAGAACAAA<br>TGTGTAAGTAACTATGCACGTCTTGACGGTACCTAATCAGAAA<br>GCCACGGCTAACTACGTGCCAGCAGCGGCGGTA	***S. epidermidis***	**99.60**
**S3**	CTGCAGTGCGGGTGCTATACATGCAGTCGAGCGAACAGACGA<br>GAAGCTTGCTCCTTCGACGTTAGCGGCGGACGGGTGAGTAACA<br>CGTGGGTAACCTACCTATAAGACTGGGATAACTTCGGGAAACC<br>GGGGCTAATACCGGATAATATTTCGAACCGCATGGTTCGATAT<br>TGAAAGATGGCTTTGCTATCACTTATAGATGGACCTGCCCCGTA<br>TTAACTAGTTGGTAAGGTAACGGCTTACCAAGGCAACCATACC<br>TAACCCAACTGAAAGGGTGATCGGCCACACTGGAACTGAAAC<br>ACGGTCCAGACTCCTACGGGAGGCAGCAGTAGGGAATCTTCCG<br>CAATGGGCGAAAGCCTGACGGAACAACGCCGCGTGAGTGATG<br>AAGGTCTTCGGATCGTAAAACTCTGTTATTAAGGAAGAACAAA<br>CGTGTAAGTAACTGTGCACGTCTTGACGGTACCTAATCAGAAA<br>GCCACGGCTAACTACGTGCCAACAGCCCCGGTAA	***S. hominis***	**95.34**

S4	CCCTGTCGCTGTCGCGCAGACGCTCGATCTCCGTAACCAGCGT CTCTTTCTCTTTCAGCAGCGCGCCCAGCTTCTCGGCGTCGCCTG ACTCCAGCAGCCGCGGTAATTTGCGGTTCAGGGCGTCCAGCTG ATCGCTCATACGTTTAATCTCGGCTTTTTCTTGCTCTTTCATGGC GAAAACTCGTTGTGGAAAACAGGCAAAGGATACACGAAAAGC GGCACAGATTCGCGCGCGTCGATGGAGCGGGGCGCCTGAATCT CAGGCGCCGGGACGTTTGAACGCCTGTTTCAGGCTGATGCGCG AGATGAGTTCAGTCAGAGAGAGCACCATGGTCGAGCGCACCA TCTGCAGATAGCGCTGTTGCTGCATGGCACGCAGCTCGGCGTC ATCGGGATGAAAGTCCGGTTTCGGCAGCCACGCAACCACACA GTGCAGCTCGCCGAACGGGCCGAGTATCTCATCGTCAGTAAAA CGGTAATCGCCCTGATCGTGATTCAGCTCTTCGCGCAGCGCCA ACAGCAGCTCGCAATCTTCATACTCATGGCGGTTGATAACGCC CAGACCGTAGATAAGCTTGAGGCGCACCGGCAGCTCGCCGAG CGGACCATCGCCCAGCAGCCGGCGGTAAT	***P. vagans***	**78.52**

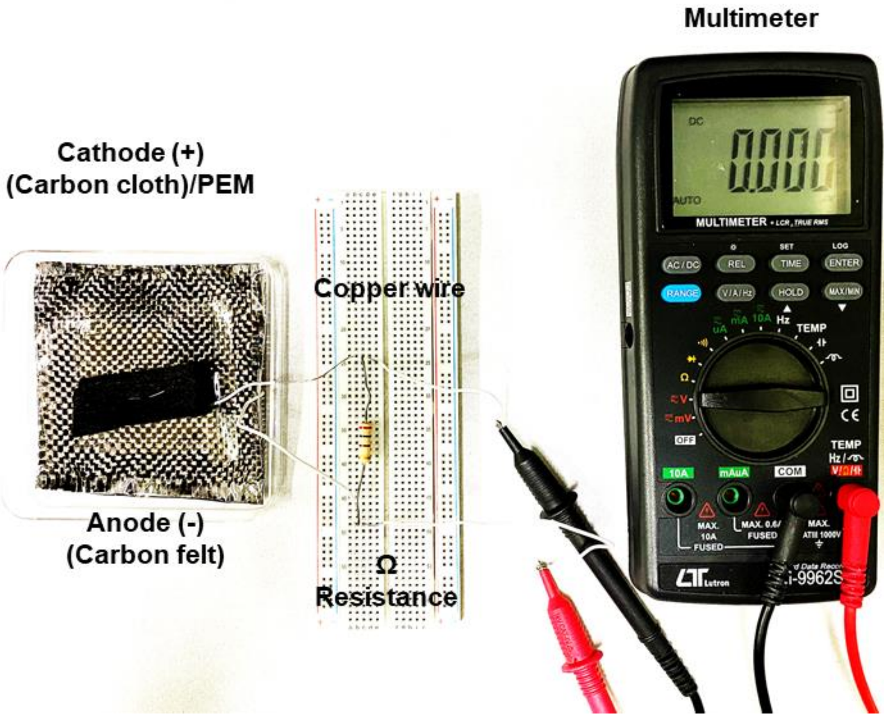

Figure S1. A photographic image of a lab-fabricated *in vitro* chamber for the detection of bacterial electricity. The components for fabrication of an *in vitro* chamber included an anode (carbon felt), a cathode (carbon cloth) which was wrapped with PEM. The carbon cloth/PEM was placed on a 10 diameter petri dish. Bacteria in media containing 2% glucose or glycerol were added on the surface of anode for electricity detection. Copper wire was used to connect anode and cathode to a 1,000 Ω resistor. The voltage change was read by a multimeter.

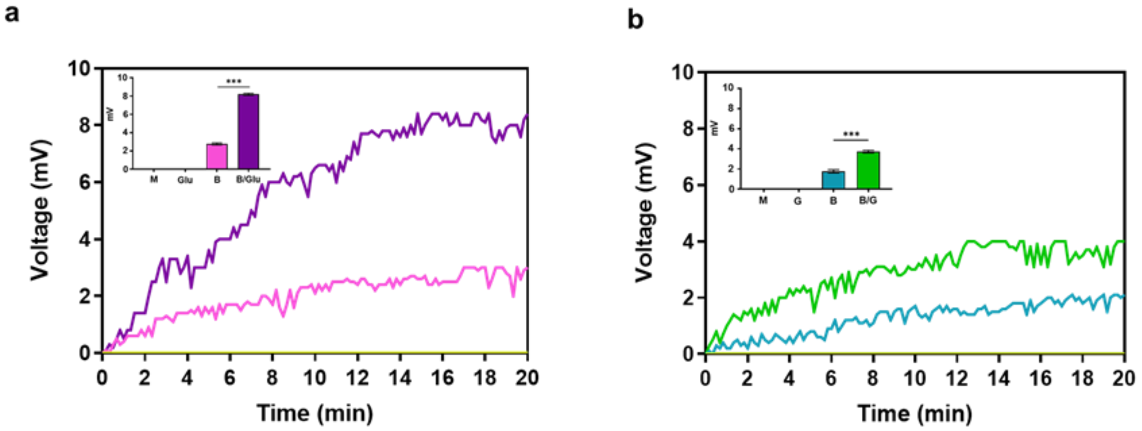

Figure S2. Electricity generation by *E. faecalis* and *S. hominis* in the presence of glucose or glycerol, respectively. (a) Media (M) or media containing glucose (Glu), *E. faecalis* (B), *E. faecalis* plus 2% glucose (B/Glu). (b) Media (M) or media containing glycerol (G), *S. hominis* (B), *S. hominis* plus 2% glycerol (B/G). Voltage changes (mV) in 20 min were measured. The inset panel represented the peak voltage of each group. Data are the mean ± SD, in triplicate of three separate experiments. ns = non-significant. ***$P < 0.001$ (two-tailed t-tests).
